# ECG data analysis to determine ST-segment elevation myocardial infarction and infarction territory type: an integrative approach of artificial intelligence and clinical guidelines

**DOI:** 10.3389/fphys.2024.1462847

**Published:** 2024-10-07

**Authors:** Jongkwang Kim, Byungeun Shon, Sangwook Kim, Jungrae Cho, Jung-Ju Seo, Se Yong Jang, Sungmoon Jeong

**Affiliations:** ^1^ Department of Medical Informatics, School of Medicine, Kyungpook National University, Daegu, Republic of Korea; ^2^ Research Center for AI in Medicine, Kyungpook National University Hospital, Daegu, Republic of Korea; ^3^ Bio-Medical Research Institute, Kyungpook National University Hospital, Daegu, Republic of Korea; ^4^ Department of Internal Medicine, School of Medicine, Kyungpook National University, Daegu, Republic of Korea; ^5^ Division of Cardiology, Department of Internal Medicine, Kyungpook National University Chilgok Hospital, Daegu, Republic of Korea

**Keywords:** ST-segment elevation detection, deep learning-based artificial intelligence, ST-segment elevation myocardial infarction, 12-lead electrocardiogram, infarction territory

## Abstract

**Introduction:**

Acute coronary syndrome (ACS) is one of the leading causes of death from cardiovascular diseases worldwide, with ST-segment elevation myocardial infarction (STEMI) representing a severe form of ACS that exhibits high prevalence and mortality rates. This study proposes a new method for accurately diagnosing STEMI and categorizing the infarction area in detail, based on 12-lead electrocardiogram (ECG) data using a deep learning-based artificial intelligence (AI) algorithm.

**Methods:**

Utilizing an ECG database consisting of 888 myocardial infarction (MI) patients, this study enhanced the generalization ability of the AI model through five-fold cross-validation. The developed ST-segment elevation (STE) detector accurately identified STE across all 12 leads, which is a crucial indicator for the clinical ECG diagnosis of STEMI. This detector was employed in the AI model to differentiate between STEMI and non-ST-segment elevation myocardial infarction (NSTEMI).

**Results:**

In the process of distinguishing between STEMI and NSTEMI, the average area under the receiver operating characteristic curve (AUROC) was 0.939, and the area under the precision-recall curve (AUPRC) was 0.977, demonstrating significant results. Furthermore, this detector exhibited the ability to accurately differentiate between various infarction territories in the ECG, including anterior myocardial infarction (AMI), inferior myocardial infarction (IMI), lateral myocardial infarction (LMI), and suspected left main disease.

**Discussion:**

These results suggest that integrating clinical domains into AI technology for ECG diagnosis can play a crucial role in the rapid treatment and improved prognosis of STEMI patients. This study provides an innovative approach for the diagnosis of cardiovascular diseases and contributes to enhancing the practical applicability of AI-based diagnostic tools in clinical settings.

## 1 Introduction

**FIGURE 1:**
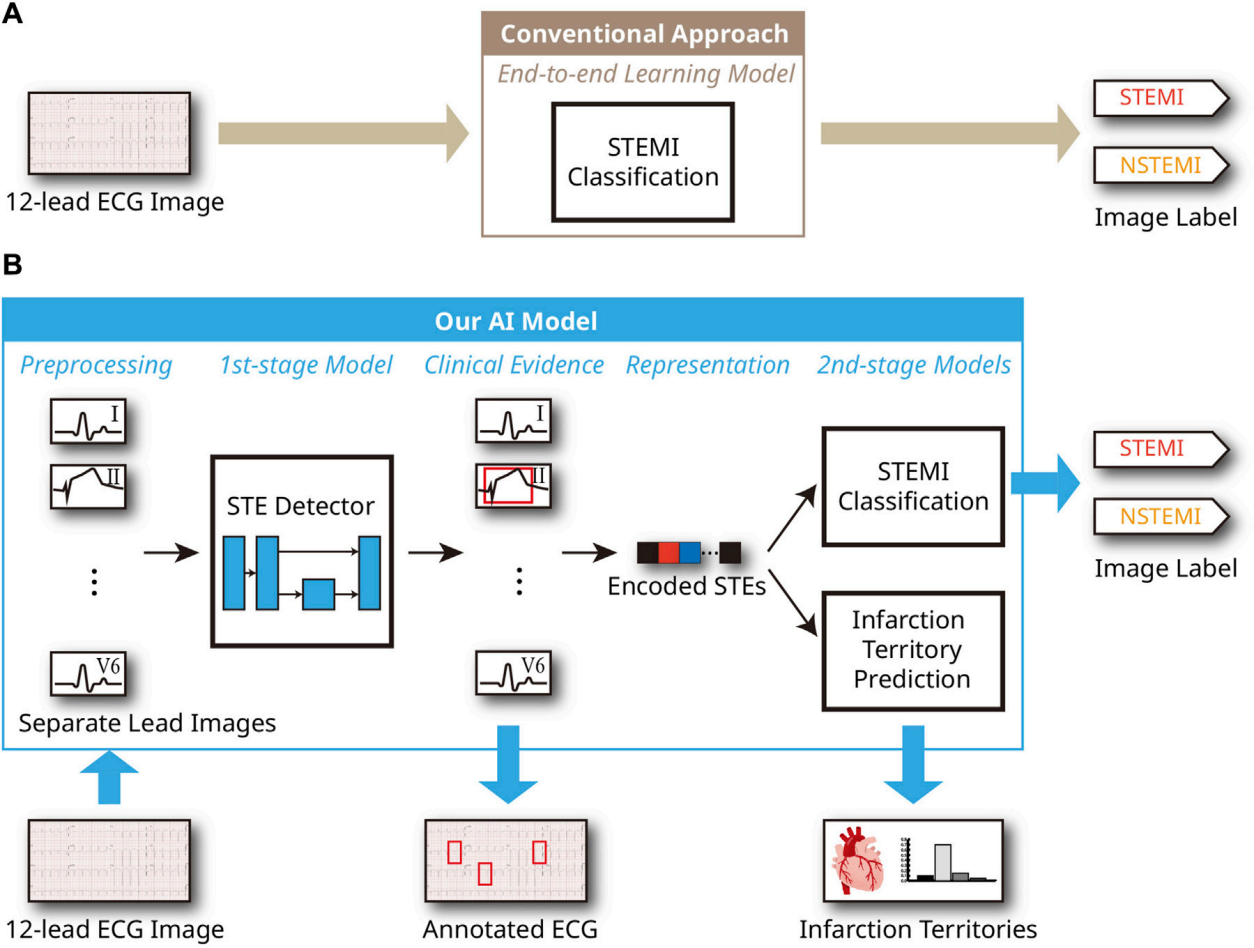
AI system process for ECG analysis from a 12-lead ECG image. **(A)** Conventional end-to-end learning approach. **(B)** Proposed model utilizing clinical evidence.

Acute coronary syndrome (ACS) is a very common cause of morbidity and mortality in the United States, with an estimated 1.5 million hospitalizations and costs of more than USD 150 billion annually, according to the American Heart Association (Kolansky, 2009). ACS includes unstable angina, non-ST-segment elevation myocardial infarction (NSTEMI), and ST-segment elevation myocardial infarction (STEMI). The pathogenesis of acute myocardial infarction involves rupture or erosion of an atherosclerotic plaque (Arbustini et al., 1999), and NSTEMI occurs in the setting of partial occlusion of the culprit coronary artery (Bhat et al., 2016). In contrast, STEMI is caused by complete occlusion of the culprit coronary artery. Thus, STEMI is more symptomatic, has a more rapid disease progression, and a higher mortality rate than NSTEMI (Rodríguez-Padial et al., 2021; Meyers et al., 2021). As such, STEMI is one of the major cardiovascular diseases with high prevalence and mortality (Benjamin et al., 2018), and timely diagnosis of STEMI is critical to reducing the risk of sudden death through prompt treatment (Murray et al., 2015). Coronary angiography (CAG) is the gold standard diagnostic method for STEMI (Wu et al., 2022). Percutaneous coronary intervention (PCI), performed through this procedure, is an effective treatment that limits the infarction size after a myocardial infarction and reduces the risk of complications and heart failure (Mehta et al., 2010; Bulluck et al., 2016). Additionally, biomarkers, cardiac imaging technologies, and electrocardiographic methods, used as auxiliary diagnostic tools, play a crucial role in diagnosing myocardial infarction (Thygesen et al., 2012). Among emergency treatment options, non-invasive ECG is the most cost-effective and irreplaceable method, allowing for continuous and remote monitoring (Siontis et al., 2021). Continuous ECG monitoring provides useful prognostic information and determines reperfusion or re-occlusion status (Thygesen et al., 2018). Therefore, it is an essential diagnostic step for suspected patients in the ambulance or hospital. Furthermore, a 12-lead ECG can be used to understand the pathogenesis of MI better and to accurately determine the location of the occluded coronary artery and myocardial infarction. Specific ECG leads can reflect various locations of the heart’s electrical activity and can distinguish different types of MI according to the area of myocardial necrosis (Meek and Morris, 2002). For example, ST-segment elevations (STEs) in leads V1, V2, V3, and V4 suggest anterior wall myocardial infarction (AMI), while STEs in leads II, III, and aVF suggest inferior wall myocardial infarction (IMI). Considering these factors, the 12-lead ECGs serve as the standard diagnostic tool for diagnosing ACS. In the clinical setting, in addition to the distinction between STEMI and NSTEMI, ECGs of STEMI patients require rapid and accurate interpretation. However, interpreting a STEMI from ECG images is challenging for ambulance paramedics, emergency physicians, and sometimes cardiologists (Debrabant et al., 2021). In previous ECG artificial intelligence (AI) studies, significant efforts were made to enhance the accuracy of STEMI and other abnormality diagnoses by leveraging deep learning for 12-lead ECG analysis (Liu et al., 2021; Ribeiro et al., 2020). These model designs are supported by evidence that AI techniques based on deep learning not only compete with but often surpass human performance in many areas, such as medical diagnostics, image and speech recognition, natural language processing, strategic game playing, algorithm discovery, and structure prediction of biomolecular interactions (Silver et al., 2016; Brown et al., 2020; Mankowitz et al., 2023; Kaufmann et al., 2023; Abramson et al., 2024). Consequently, those studies attempting to diagnose heart diseases have demonstrated that these techniques yield meaningful results in practical 12-lead ECG settings. However, these existing studies have only conducted experiments on 1-dimensional digital ECG sequence data directly obtained from devices and not ECG images derived from printed papers, although printed versions are often considered the universal format for ECGs. In contrast, the literature on conventional image processing techniques for ECGs from printed papers includes numerous efforts to digitize these images into sequences, apply geometric transformations, and recognize specific patterns (Lence et al., 2023; Badilini et al., 2005; Waits and Soliman, 2017). However, most of these approaches struggle with noise sources such as printing errors, poor skin-electrode contact, and other interferences, resulting in insufficient accuracy and performance that is not competitive with human professionals, such as emergency medicine residents or cardiologists. Given the complexity of these noises and the real-world contexts, the data-driven, end-to-end learning capabilities of deep learning-based AI are essential. The conventional deep learning approach to perform STEMI classification is illustrated in Figure 1A. To our knowledge, no study has yet demonstrated the clinical significance of interpreting and classifying STEMI through deep learning in 12-lead ECG images. This study presents a clinically-grounded and interpretable AI model for STEMI ECG analysis. This AI model aims to distinguish between STEMI and NSTEMI and accurately predict infarction territory on STEMI ECGs, serving as an auxiliary tool for clinicians to facilitate rapid diagnosis and treatment. Figure 1B illustrates the structure of the proposed model. Initially, it uses a deep learning-based algorithm to detect ST-segment elevations in each lead of the 12-lead ECG image through supervised learning (Rashidi et al., 2019), providing clinical evidence for further analysis. It then distinguishes between STEMI and NSTEMI and, if classified as STEMI, identifies the infarction territory based on the detected STEs. The detailed process is provided in the Materials and Methods section. The Experiments and Results section evaluates the effectiveness of our approach and compares its performance to conventional deep learning approaches, illustrated in Figure 1A.

## 2 Materials and methods

### 2.1 Data collection

#### 2.1.1 Dataset

**FIGURE 2:**
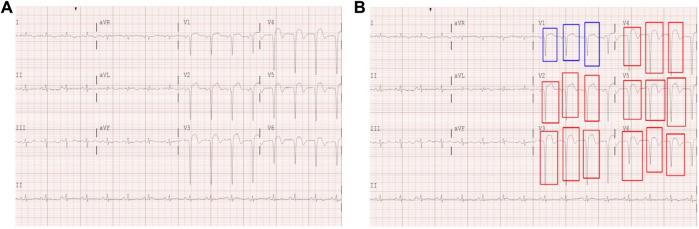
**(A)** Example of a digitized 12-lead ECG image. **(B)** Hand-drawn STE (red) and lesser STE (blue) box annotations on a 12-lead ECG image.

A standard supine 12-lead ECG of 25 mm/s and 10 mm/mV was used. We selected the first ECG for patients with MI when they visited the emergency department for the MI ECG. The Philips ECG system (Philips Medical System, PageWriter TC30, TC70) was used for the baseline ECG data. The dataset consists of ECG images, of which the ECG region consists of a grid-shaped background and a signal portion, as shown in Figure 2A. The signal part of the ECG area comprises thin lines. We collected ECG images of 888 MI patients, including 677 ST-segment elevation myocardial infarction (STEMI) patients and 211 non-ST-segment elevation myocardial infarction (NSTEMI) patients, recorded in the emergency department of Kyungpook National University Hospital from January 2011 to December 2019. Table 1 provides the distribution of samples for STEMI and NSTEMI datasets. It should be noted that the total number of samples for infarction territory (872) exceeds the number of STEMI samples (677). This discrepancy is because a single STEMI patient can have multiple coronary occlusions. The KNUH Institutional Review Board approved the use of this dataset (2020-12-022-011).

**TABLE 1:** Distribution of samples for STEMI and NSTEMI datasets.

Description	Counts
STEMI	677
Anterior MI	467
Lateral MI	233
Inferior MI	123
Suspected left main disease	49
NSTEMI	211
Total	888

#### 2.1.2 Data annotation

This study performed model learning using a supervised learning approach. For the 12-lead ECG images, the STEMI and NSTEMI diagnostic labeling was classified based on medical records and the clinical judgment of physicians. In addition to ST-segment elevation, we annotated areas of mild ST elevation (i.e., lesser STE) to consider these abnormalities as potential indicators to distinguish STEMI and NSTEMI cases in ECGs. To generate ground truth labels, human encoders manually annotated the STE (a box where the ST segment rises more than 1 mm, including the QRS complex) and lesser STE (a box where the ST segment rises 1 mm or lesser, including the QRS complex) for each lead of the 12-lead ECG images using a Python package called labelImg (GitHub, 2015) (Figure 2B). The criteria for ST elevation at the J point was based on the American College of Cardiology/American Heart Association STEMI guidelines, defined as an elevation of 1 mm or more above the baseline at the J point. All labels and annotations in the STEMI and NSTEMI ECG annotation dataset were performed using the gold standard of three board-certified cardiologists who agreed on the annotation placement through discussion. This study was retrospective and did not require informed consent.

### 2.2 Data preprocessing

The original ECG data were available in printed papers, and the scanned ECG region had a resolution of 1,228 × 2,460. While we used 12-lead ECGs, there are 13 different figures because lead II (L2) has one more recording of longer duration, whereas the other channels have only short recordings, as shown in Figure 2A. For simplicity, we split the long L2 recording into four evenly divided subregions and detected ST elevations separately in each subregion. We did not use other clinical information, such as sex or age, which can be obtained from the ECG reports.

### 2.3 Model architecture

In this study, we first detect regions of STE and lesser STE patterns from the given ECG, and two inference procedures were conducted based on the detection result: STEMI classification and prediction of infarction territory. The brief architecture of the model is illustrated in Figure 1.

#### 2.3.1 Detection of ST-segment elevations

**FIGURE 3:**
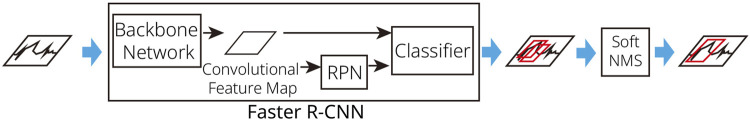
ST-segment elevation detection procedure.

As described in subsection 2.2, the only preprocessing required is the manual separation of image regions for each ECG lead, performed once before data processing begins. These clipped images from the original ECG serve as input for our detector models. To detect STEs, we trained a Faster R-CNN model (Ren et al., 2016) with ResNet-50 (He et al., 2016) serving as the backbone network. Faster R-CNN is an object detection algorithm and its Region Proposal Network (RPN) is responsible for generating region proposals for detections. The algorithm begins by processing the input image through a Convolutional Neural Network (CNN) to generate feature maps, which capture important visual information from the image. Next, Faster R-CNN employs a RPN to suggest potential object locations. The RPN predicts the likelihood of an object being present in each proposed region and provides the coordinates of the corresponding bounding boxes. During this process, Faster R-CNN performs bounding box regression to refine these proposals, aiming to accurately predict the locations of objects by minimizing the difference between the predicted bounding boxes and the actual ground truth boxes. Finally, for each refined bounding box, the algorithm predicts the object class, resulting in the final detection output. Through this integrated approach, Faster R-CNN achieves high accuracy and speed in object detection. Thus, Faster R-CNN is notable for its efficient region proposal mechanism and its focus on accurately predicting both the locations and classes of objects within an image. As we described earlier, we utilized pre-trained model weights of ResNet-50 for constructing the convolutional feature maps of the Faster R-CNN, instead of random initialization. In this study, the RPN of Faster R-CNN applies a 3 × 3 convolutional filter to generate region proposals using anchor boxes of various sizes. ResNet-50, the backbone network for Faster R-CNN, consists of 50 layers and employs residual (shortcut) connections that skip over one or more layers. During the training phase of Faster R-CNN, ground-truth bounding boxes of STEs and lesser STEs, annotated by cardiologists, are provided as labels. Using these annotations, the model is trained, enabling it to generate accurate bounding boxes during inference. During bounding box regression of Faster R-CNN (Ren et al., 2016), bounding box information is encoded as shown in Equation 1.

$$t_{x}=\frac{x-x_{a}}{w_{a}},t_{y}=\frac{y-y_{a}}{h_{a}},t_{w}=\log\frac{w}{w_{a}},t_{h}=\log\frac{h}{h_{a}},$$ (1)

where *x*, *y*, *w*, and *h* represent x-y coordinates of the center of a bounding box and its width and height, respectively, while *a* denotes the anchor index. Outputs of Faster R-CNN detectors are predicted bounding boxes of detected objects (STE and lesser STE). Since the raw outputs of the Faster R-CNN detector often include multiple overlapping bounding boxes that are not semantically distinct, to refine these results, we additionally apply soft non-maximal suppression (soft NMS) (Bodla et al., 2017). We determined optimal threshold values for soft NMS filtering by systematically evaluating Intersection over Union (IoU) and score threshold values from 0.0 to 1.0 in increments of 0.1. Finally, an IoU threshold of 0.5 and a score threshold of 0.7 were selected as they provided the best results on the validation dataset. The procedure of the STE detection is illustrated in Figure 3. Notably, our approach can directly identify STE and lesser STE regions from scanned ECG paper images, eliminating the need for additional preprocessing steps like grid removal or smoothing.

#### 2.3.2 Classification of STEMI

For automated classification of STEMI, various methods exist to encode information detected from an image. In this study, we employed the following information encodings: (1) the counts of detected STEs and lesser STEs from the given image; (2) the counts of detected STEs for each lead; (3) the counts of detected STEs and lesser STEs for each lead; (4) the presence of detected STEs and lesser STEs from the given image; (5) the presence of detected STEs for each lead; (6) the presence of detected STEs and lesser STEs for each lead. We denote these six feature encoding schemes, as shown in Table 2. For example, if four STEs and one lesser STE are detected at the first lead, and two lesser STEs are at the second lead, then the representations will be: (Image-Detailed Count) [4, 3]; (Lead-STE Count) [4, 0, 0, …, 0]; (Lead-Detailed Count) [4, 1, 0, 2, 0, 0, …, 0]; (Image-Detailed Presence) [1, 1]; (Lead-STE Presence) [1, 0, 0, …, 0]; and (Lead-Detailed Presence) [1, 1, 0, 1, 0, 0, …, 0]. For these six cases, classification performances are separately evaluated in the results for comparison. As a classification model, weighted ensemble models (Erickson et al., 2020) were trained to classify STEMI from the STE detection result. Although we tested classifiers such as Support Vector Machines and Random Forests, the weighted ensemble model consistently outperformed the others. Therefore, we selected the weighted ensemble model for STEMI classification. The weighted ensemble model consists of k-nearest neighbors, LightGBM, CatBoost, XGBoost, RandomForest, ExtraTrees, and Multi-layer Perceptron. Comparisons of the encodings are presented in Table 5 in the Results subsection.

**TABLE 2:** Encoding method for STE detection results.

Encoding method	Description
Image-Detailed Count	Counts of detected STEs and lesser STEs from an image
Lead-STE Count	Counts of detected STEs for each lead
Lead-Detailed Count	Counts of detected STEs and lesser STEs for each lead
Image-Detailed Presence	Presence of detected STEs and lesser STEs from an image
Lead-STE Presence	Presence of detected STEs for each lead
Lead-Detailed Presence	Presence of detected STEs and lesser STEs for each lead

#### 2.3.3 Prediction of infarction territory

In this subsection, we describe a method for identifying infarction territory in patients with STEMI. Using detection results of STEs on 12 leads as features, we used a weighted ensemble model to predict vascular problems. The areas of infarction were classified into four regions: anterior, lateral, inferior, and suspected left main disease. We referenced the clinical criteria in Table 3 to identify infarction territory based on ECG findings.

**TABLE 3:** Clinical criteria for identifying infarction territories on the ECG.

Infarction territory	Condition
Anterior	More than one lead shows STE among V1, V2, V3, and V4
Lateral	Two or more leads show STE among V5, V6, L1, and aVL
Inferior	Two or more leads show STE among L2, L3, and aVF
Suspected left main disease	STEs are observed in aVR

We applied these clinical criteria to the AI model, conducting a process in which the STE Detector predicted infarction territory based on anticipated annotations. The predicted results were then compared and analyzed for similarity with the target labels classified by the actual infarction territories. A classifier using weighted ensembles learns the relationships between the encodings of detected STEs (and lesser STEs) and the target territories.

## 3 Experiments and results

### 3.1 Experiment setup

To implement the proposed system, we used deep learning libraries and frameworks such as AutoGluon, MMDetection, and PyTorch (Erickson et al., 2020; Chen et al., 2019; Paszke et al., 2019). Specifically, we used MMDetection for Faster R-CNN implementation, and PyTorch for the baseline models, including ResNet-50, Inception-ResNet-v2 (Szegedy et al., 2017), and ConvNeXt (Liu et al., 2022). For the classification of STEMI in section 2.3.2, we used AutoGluon’s implementations of LightGBM, CatBoost, XGBoost, RandomForest, ExtraTrees, and Multi-layer Perceptron. A computing server with three NVIDIA A10 Tensor Core GPUs with 24 GB memory is used in the experiments. The total training time for a model is approximately 9 h on the server, and a 5-fold cross-validation process, which takes around 45 h. For inference, processing a single ECG sample takes about 500 milliseconds. All performance metrics are obtained by averaging the results from 5-fold cross-validation. To compare the performance of the proposed method with end-to-end AI models consisting of a single classification stage (i.e., without explicit detection of STEs), we fine-tuned deep learning models such as ResNet-50, Inception-ResNet-v2, and ConvNeXt using the provided ECG dataset. The pre-training was conducted using the Microsoft COCO dataset (Lin et al., 2014), a large-scale dataset widely used for object detection and segmentation tasks. The learning rate was set to 0.001 for 100 epochs of training, and an early stopping criterion was applied to avoid overfitting.

### 3.2 Results

In this subsection, we present performance evaluations for the detection of STE, the classification of STEMI, and the identification of infarction territories.

In each ECG image, there were 16 subregions corresponding to the leads. In each subregion, the detector attempts to generate bounding boxes for STE or lesser STE patterns. The average performance of detecting ST-segment elevations and their standard deviations is presented in Table 4. The experiment was conducted using a 5-fold cross-validation technique. Sensitivity scores are slightly higher than precision, which is beneficial for the detector, as false negatives are generally riskier in medical situations.

**TABLE 4:** STE detection performances from 5-fold cross-validations.

Target	Ground truths	Detections	Sensitivity	Average precision
STE	1,414.6 (75.9)	1,350.8 (76.2)	0.825 (0.020)	0.794 (0.020)
Lesser STE	1,441.2 (108.0)	1,570.8 (181.1)	0.814 (0.024)	0.734 (0.029)

Table 5 describes the STEMI detection performances with various STE encoding schemes. As our initial assumption that minor abnormalities, such as lesser STE, can also provide a hint to STEMI, feature encodings with both STE and lesser STE outperformed other encodings. However, the superior performance of *Presence* encodings over *Count* encodings was difficult to anticipate and requires further interpretation. This outcome can be interpreted from the following perspective. In the context of STEMI classification, the rich information provided by Count encodings might not be as critical because clinical decisions are generally unaffected by whether there are five or six STEs. Instead, the simplicity of the feature space in Presence encodings offers an advantage over the richness of Count encodings for training AI models, as a more complex feature space typically requires more data samples to achieve similar generalization performance. In this experiment, large-scale training data were unavailable; therefore, for the STEMI classification task, Presence encodings may be more effective than Count encodings. As a result, the Lead-Detailed Presence encoding proved to be the most effective encoding for STE detection in the STEMI classification task.

**TABLE 5:** Performance of STEMI classification from 5-fold cross-validations.

Encoding	AUROC	AUPRC	Accuracy	Sensitivity	Specificity	Precision	F1-score
Image-Detailed Count	0.878	0.940	0.891	0.925	0.782	0.930	0.927
Lead-STE Count	0.775	0.873	0.875	**0.973**	0.576	0.876	0.922
Lead-Detailed Count	0.907	0.953	**0.899**	0.916	0.841	0.948	**0.931**
Image-Detailed Presence	0.926	0.971	0.872	0.863	0.901	0.964	0.910
Lead-STE Presence	0.925	0.969	0.871	0.855	**0.918**	**0.970**	0.909
Lead-Detailed Presence	**0.939**	**0.977**	0.895	0.894	0.897	0.965	0.927

To compare our approach with other deep learning models, we conducted fine-tuning procedures for ResNet-50, Inception-ResNet-v2, and ConvNeXt and evaluated their performance on STEMI detection. These models perform end-to-end learning and one-stage STEMI detection, meaning they do not explicitly detect STE or lesser STE regions but instead provide a direct decision for the given image. In contrast, our approach consists of a two-stage process: explicit detection of STEs, followed by STEMI classification based on the information from the detected STEs. By explicitly detecting STEs, our model incorporates expert knowledge from cardiologists during the model construction phase, resulting in superior performance compared to one-stage deep learning models, as shown in Table 6. Furthermore, in the inference phase, our model provides explicit detection results to assist medical clinicians in their decision-making process, enhancing the explainability of the model and avoiding the limitations of a black-box approach.

**TABLE 6:** Comparison to other deep learning models.

Model	AUROC	AUPRC	Accuracy	Sensitivity	Specificity	Precision	F1-score
ResNet-50	0.780	0.925	0.656	0.578	**0.909**	0.954	0.719
Inception-ResNet-v2	0.739	0.911	0.613	0.535	0.864	0.927	0.679
ConvNeXt	0.594	0.815	0.723	0.859	0.409	0.824	0.841
Our Work	**0.939**	**0.977**	**0.895**	**0.894**	0.897	**0.965**	**0.927**

**FIGURE 4:**
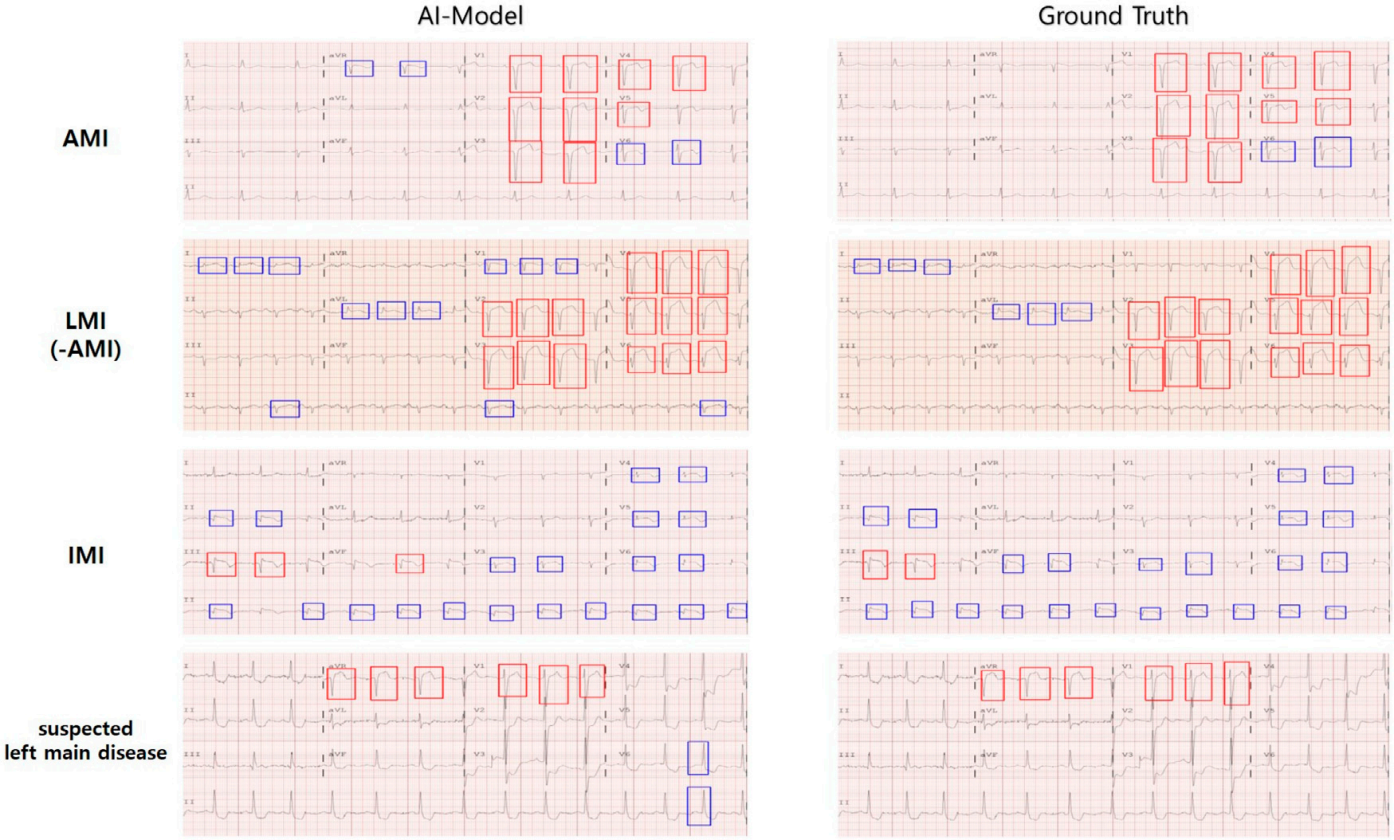
Cardiologists provided labels for infarction territories and annotated STE in 12-lead ECGs, and the AI model predicted the area of infarction and detected STE in 12-lead ECGs. The color of the annotation boxes for STE (and lesser STE) is as follows: STE is red, lesser STE is blue.

Based on the STE and lesser STE detection results, we trained and tested weighted ensemble models, and Table 5 illustrates the average and standard deviations of performances obtained from 5-fold cross-validation. As shown in Table 7, the performance of suspected LM prediction was much worse than predictions of other territories, and it is partly rooted in the fact that the suspected left main disease has much fewer samples than the others, as described in Table 1. We also visualized the images to enhance our understanding of these experimental results, as shown in Figure 4.

**TABLE 7:** Infarction territory identification performances from 5-fold cross-validations.

Territory	AUROC	Accuracy	Sensitivity	Specificity	Precision	F1-score
Anterior	0.955 (0.015)	0.919 (0.023)	0.940 (0.025)	0.895 (0.045)	0.91 (0.032)	0.924 (0.021)
Lateral	0.925 (0.047)	0.898 (0.037)	0.874 (0.079)	0.906 (0.039)	0.769 (0.090)	0.816 (0.073)
Inferior	0.966 (0.039)	0.954 (0.022)	0.904 (0.103)	0.962 (0.029)	0.811 (0.108)	0.848 (0.064)
Suspected left main disease	0.838 (0.083)	0.924 (0.052)	0.767 (0.133)	0.934 (0.050)	0.450 (0.150)	0.559 (0.149)

## 4 Discussion

STEMI is classified as the most lethal form of coronary artery disease. It is caused by a complete or near-complete blockage of a coronary artery that supplies blood to the heart muscle, which can lead to extensive damage to the myocardial tissue. Rapid and accurate diagnosis of STEMI can significantly improve patient survival and recovery. However, inappropriate and false-positive activation in the cardiac catheterization laboratory was reported to be as high as 2.7% and 20%, respectively (Rokos et al., 2010), which was highlighted as a major problem in the diagnostic and treatment process. Moreover, the uneven distribution of diagnostic levels and medical resources increases the possibility of misdiagnosis and missed diagnosis of STEMI, a major obstacle in providing patients with optimal diagnostic and treatment strategies. Electrocardiogram is an objective, cost-effective, and widely used tool for diagnosing STEMI. An ECG records the electrical activity of the heart, which allows for identifying signs of coronary artery occlusion and myocardial damage. However, ECG interpretation is highly dependent on the experience and judgment of the physician, which can lead to a lack of diagnostic consistency, a time-consuming and labor-intensive process.

An ECG-based STEMI diagnosis using artificial intelligence technology represents a significant step forward in addressing these issues. AI can learn patterns from large datasets and diagnose STEMI quickly and accurately based on these patterns. This is important in reducing the door-to-balloon (D2B) time, with shorter D2B time associated with lower in-hospital mortality and 6-month mortality (Nallamothu et al., 2015). Reducing D2B time helps to minimize damage to the heart muscle, increase the possibility of recovery of cardiac function, and improve long-term survival. The results of this study suggest that AI, especially CNN deep learning models, can be effectively used to analyze each lead by detection in a 12-lead ECG. We built an ECG database of 888 MI patients in a real hospital emergency department. We used 5-fold cross-validation in all experiments to prevent the model from overfitting to the training data and improve its generalization ability. We were able to use the data efficiently because all data samples can be used for training and validation. In addition, by evaluating the model using multiple data sets, we increased the stability and reliability of the model, as the performance of the model does not depend on a specific part of the data. Our STE detector accurately detected STE and lesser STE with an average mAP of 0.76 in each of the 12 leads. When applied to the AI model, it showed high classification performance with an average AUROC of 0.939 and AUPRC of 0.977 for STEMI and NSTEMI, respectively. In addition, the ECG showed a significantly high sensitivity for the detection of anterior MI, recording an average ACC (0.91), SEN (0.94), SPEC (0.89), F1-score (0.92), and AUROC (0.95). Moreover, the AI model demonstrated accurate performance even for lateral MI and inferior MI, which are more challenging to interpret on ECG than anterior MI. The average scores were ACC (0.89, 0.95), SEN (0.87, 0.90), SPEC (0.90, 0.96), F1-score (0.81, 0.84), and AUROC (0.92, 0.96). Even for the relatively infrequent suspected left main disease, it achieved high performance with ACC (0.92), SEN (0.76), SPEC (0.93), and AUROC (0.83).

The main contribution of this study is to demonstrate that our AI model excels in classifying STEMI and NSTEMI and identifying infarct locations. Previous studies reported that the classification performance of AI for STEMI had average AUROC, ACC, SEN, and SPEC values of 0.99, 0.98, 0.95, and 0.99, respectively, highlighting the superiority of AI models in classifying STEMI and NSTEMI ECGs (Wu et al., 2022; Chen et al., 2022; Zhao et al., 2020). However, from a patient care support perspective, the decisions made by AI did not provide clear evidence for ST segment elevation, a critical indicator for STEMI diagnosis in reliable ECGs. To address this issue, we utilized an ST segment elevation (STE) detector to provide clinically interpretable ECG analyses to clinicians, demonstrating the detector’s excellent performance in identifying STE and lesser STE across 12 ECG leads. Notably, while our STE detection based on clinical guidelines had the AUROC, ACC, SEN, and SPEC values (0.939, 0.895, 0.894, 0.897) relatively lower than those of previous AI models, it improved the reliability of AI judgments by effectively distinguishing STEMI and NSTEMI at a level comparable to that of cardiologists (Wu et al., 2022; Zhao et al., 2020) and visualized the ability to classify infarct locations similarly to cardiologists (Figure 4). Additionally, it provides more detailed information about the anatomical key locations of ST segment elevation myocardial infarction, which differentiates our study from previous research.

The significance of this work goes beyond the advancement of AI technology. We explored ways to improve the depth and accuracy of clinical information that AI models can provide through ECG analysis. Furthermore, this work demonstrates the potential of medical evidence-based AI developed through close collaboration between AI developers and healthcare professionals to be usefully applied in real clinical settings. Such collaboration will make AI technology a clinically interpretable and reliable tool for healthcare professionals, ultimately leading to better patient outcomes.

However, several obstacles need to be overcome before the results of this study can be applied to actual clinical practice. First, there is a need for data standardization between hospitals. It is difficult to guarantee the compatibility of AI tools, as the data format or structure varies depending on the type or manufacturer of the ECG machine used in each hospital, making it difficult to apply AI tools to the clinical field. It is necessary to ensure technical compatibility through standardization of data between hospitals, and system integration between medical institutions and manufacturers is required for this. Secondly, enhancing the understanding of AI technology among medical personnel presents a significant challenge. Utilizing these tools can be difficult for those with limited knowledge of AI. An initial solution might involve carefully considering the UI design of these tools to accommodate non-AI experts better. However, providing additional promotion and education is crucial for medical personnel to use AI tools effectively (Kim et al., 2024). Such measures will encourage the adoption of AI tools across multiple centers and promote a safer and more standardized medical environment. Third, AI-based ECG diagnostic algorithms must be rigorously validated across diverse racial groups to address and minimize racial biases and ensure fairness and accuracy. Fourth, the classification performance of AI should be thoroughly assessed, including comparisons with a control group without a history of acute coronary syndrome (ACS). This evaluation should extend to detecting early phases of STEMI by including lesser ST elevations alongside significant ones, Additionally, considering rare cases that may be observed in STEMI, such as tall T waves and Q wave abnormalities, would enhance the clinical effectiveness of the proposed STEMI classification AI model, which requires further exploration. Fifth, while this study’s retrospective single-center design has provided initial insights, its generalizability is limited. Despite rigorous internal validation through cross-validation, additional studies involving external validation in varied settings are essential to enhance reliability and applicability. Nevertheless, this study employed ECG machines commonly used in clinical practice and favored digitized ECG images over raw data to improve the generalizability of the findings. However, there is still potential to further enhance the method’s performance by leveraging the extensive information available from the raw digital signals recorded by these devices. As a part of our future work, we aim to explore data augmentation using generative models that facilitate domain transfers between raw signals and printed ECG images. Additionally, we will consider detecting other visual cues, such as peaks or QRS complexes, to enrich the feature space (Ciusdel et al., 2020). For instance, heartbeat information derived from peak detection could be incorporated into the feature vector or used to calculate additional features, such as the STE ratio normalized by cardiac cycles. Furthermore, although we measured the performance of the methods using cross-validation, there remains a need to investigate the effects of hyper-parameter variations. To gain a deeper understanding and improve robustness to hyper-parameters, we plan to conduct an ablation study. Lastly, we will perform experiments to thoroughly test the robustness of the proposed model, including its behavior in edge cases. For instance, in some cases, an ECG plot may overlap with the region of another lead, causing two signal lines to merge or become difficult to distinguish. It is crucial to observe how the model responds to such abnormal cases to ensure its applicability in real-world scenarios.

In conclusion, effective use of AI for ECG interpretation requires technical development, a supportive institutional structure, and an educational system. This integrated approach will guide future research directions in this study and play a crucial role in expanding the use of AI in ECG analysis. Such advancements will aid medical professionals by providing direct support in clinical settings, boosting their confidence in the performance of AI. The role of AI as a co-pilot in facilitating quick and accurate diagnoses can be critical in saving lives, especially in emergencies. Since the direct diagnosis is still made after review by a cardiologist, patients will be free from anxiety and prejudice about AI. This is expected to make a significant contribution to the resolution of the uneven distribution of medical resources and the improvement of patient outcomes.

## 5 Conclusion

The present study evaluates an AI model for STEMI diagnosis and identifying infarction territories from 12-lead ECG images, integrating AI and clinical guidance. Two significant benefits are obtained by explicitly guiding the detection of ST-segment elevations.

First, it addresses the challenge of insufficient training data. Without clinical guidance, the model would struggle to identify effective features from large-scale data, which are often scarce and expensive to acquire and annotate in medical data analysis. The model can focus on relevant features even with a limited dataset by incorporating clinical evidence during the training phase.

Second, the model offers explainability, aiding clinical practitioners, including cardiologists, in understanding its diagnostic process. By providing clinical evidence during the inference phase, clinicians can make prompt decisions in urgent situations and evaluate the reliability of the model’s outputs, such as STEMI classification and identifying infarction territories.

The study demonstrates the model’s effectiveness in diagnosing STEMI and identifying infarction territories. The proposed model performs superiorly compared to conventional end-to-end learning AI approaches.

In summary, this study highlights the potential of AI models enhanced with clinical evidence and developed through close collaboration between AI developers and cardiology specialists. Such implementation ensures that AI technology becomes a clinically interpretable and reliable tool, ultimately improving patient outcomes.

## Data Availability

The original contributions presented in the study are included in the article/supplementary material, further inquiries can be directed to the corresponding authors.
